# High postoperative monocyte indicates inferior Clinicopathological characteristics and worse prognosis in lung adenocarcinoma or squamous cell carcinoma after lobectomy

**DOI:** 10.1186/s12885-018-4909-1

**Published:** 2018-10-22

**Authors:** Yang Hai, Nan Chen, Wenwen Wu, Zihuai Wang, Feng Lin, Chenglin Guo, Chengwu Liu, Weimin Li, Lunxu Liu

**Affiliations:** 1 Department of Thoracic Surgery, West China Hospital, Sichuan University, Address: No. 37, Guoxue Alley, Chengdu, 610041 Sichuan China; 2 West China School of Medicine, Sichuan University, Chengdu, 610041 China; 3 Department of Respiratory and Critical Care Medicine, West China Medical School/West China Hospital, Sichuan University, Chengdu, China

**Keywords:** Lung squamous carcinoma, Lung adenocarcinoma, Peripheral monocyte count, Prognosis

## Abstract

**Background:**

Peripheral monocyte count is an assessable parameter. Recently, evidence suggested an elevated preoperative monocyte counts predicting poor prognosis in malignancies. The aim of this study was to determine the prognostic effect of early postoperative blood monocyte count in patients with lung adenocarcinoma or squamous cell carcinoma following lobectomy.

**Methods:**

We retrospectively reviewed patients with operated lung adenocarcinoma or squamous cell carcinoma from 2006 to 2011 in Western China Lung Cancer database. Univariate analysis on disease-free survival (DFS) and overall survival (OS) was performed using the Kaplan-Meier and log-rank tests, and multivariate analysis was conducted using the Cox proportional hazards regression model.

**Results:**

There were 433 patients enrolled in our analysis. High postoperative elevated monocyte was associated with male gender (*P* < 0.001), positive smoking history (*P* = 0.005), and higher N stage (*P* = 0.002) and higher tumor stage (*P* = 0.026). Two-tailed log-rank test indicated patients with an early postoperative elevated monocyte count predicted a poor DFS and OS overall (*P* < 0.001, *P* < 0.001, respectively) as well as in subgroup analysis, and further presented as a promising independent prognostic factor for both DFS and OS (HR = 2.991, 95%CI: 2.243–3.988, *P* < 0.001; HR = 2.705, 95%CI: 1.977–3.700, *P* < 0.001, respectively) on multivariate analysis. However, no significance was detected for preoperative monocyte in multivariate analysis.

**Conclusions:**

Elevated early postoperative peripheral monocyte count was an independent prognostic factor of poor prognosis and inferior clinicopathological features for patients with operable lung adenocarcinoma or squamous cell carcinoma by lobectomy.

## Background

Lung cancer is the leading cause of cancer-related death worldwide, with 5-year survival rates of less than 17% [1]. Non-small-cell lung cancer (NSCLC) accounts for approximately 85% of all lung cancers. NSCLC is further subtyped into adenocarcinoma and squamous cell carcinoma, which respectively account for approximately 40% and 25–30% of lung cancers [1, 2]. If operable, surgery provides the best chance to cure NSCLC [3]. There is a need for a comprehensive perioperative evaluation system for this deadly disease.

The interplay between cancer and inflammation was postulated back in the late nineteenth century, and even today continues to be an active area of research. [2]. Such research often focus on the effects of tissue necrosis factor (TNF), interleukin (IL)-1, IL-6, matrix metalloproteinases, vascular endothelial growth factor, etc. However, peripheral blood cells, monocytes/macrophages, neutrophils, dendritic cells, and natural killer cells form the first line of immune defense in normal situations [4]. Recently, elevated preoperative monocyte counts have recently been shown to predict poor prognosis in various types of malignancies, including hepatic cell carcinoma, malignant lymphomas as well as lung adenocarcinoma [5–7]. However, it is still unclear if an elevated early postoperative monocyte count was associated with a poor prognosis in lung adenocarcinoma or squamous cell carcinoma after lobectomy.

We focused on the early postoperative period because it is the most turbulent stage of the host immune system which may be caused by the surgical trauma and/or tumor removal effect, and we hypothesized that this change in immune environment is related with tumor progression. The aim of this study was to evaluate whether an elevated early postoperative monocyte count predicted a poor prognosis in patients with operable lung adenocarcinoma or squamous cell carcinoma after lobectomy.

## Methods

### Study population

The data were retrieved from Western China Lung Cancer database (WCLC). We enrolled patients with operable lung adenocarcinoma or squamous cell carcinoma treated with lobectomy at West China Hospital, Sichuan University between 2006 and 2011. All patients were > 18 years of age, with complete clinicopathological data, and proven to be lung adenocarcinoma or squamous cell carcinoma after surgery. Preoperative evaluation included physical examination, blood routine examination, tumor markers test, chest X-ray and computed tomography, brain magnetic resonance imaging (MRI), bone scintigraphy, and bronchoscopy and integrated positron emission tomography scan and CT (PET/CT) scan when necessary. The eligibility criteria were: 1) lobectomy with no microscopic residual tumor; 2) no preoperative chemotherapy and/or radiotherapy; 3) no previous history of other malignancies; 4) no evidence of infections such as pneumonia; 5) availability of laboratory data and follow-up information. All peripheral venous blood samples were collected from patients within 1 week before and 4 days after surgery. If multiple post-operative blood samples were drawn, only the first sample after surgery was used for analysis. Absolute peripheral blood count and the percentage were analyzed for each blood sample. Histological classification was made with reference to the latest WHO guideline [8]. The stages of lung cancer were confirmed based on the 7th edition of TNM classification of malignant tumors [9]. Study approval was granted by the Institutional Review Board at the West China Hospital, Sichuan University.

### Evaluation of clinicopathological factors

Baseline characteristics included age, sex, underlying diseases, smoking history, pathological stage, pathological tumor status, pathological lymph node status, and peripheral blood counts and the percentages. Blood samples analysis were performed within the clinical laboratory of our hospital with a Cell-Dyn 3700 (Abbott Diagnostics, USA). Other data included the surgical date and procedures.

### Treatment and follow-up

Lobectomy was performed on all patients with intent to cure, and systematic nodal dissection was carried out. The resection was done both macroscopically and histologically with a negative tumor margin and no evidence of distant metastasis. Patients were regularly followed up at outpatient department 1 month after surgery, every 3 months for the first year, every 6 months for the next 4 years, and once annually thereafter. Patients received a physical examination, blood routine examination, chest and brain and upper abdomen CT scan at each follow-up. Bone scintigraphy was performed every 12 months. In particular, all patients treated in our department received phone call follow-up regularly, during which we recorded their living status, tumor recurrence/metastasis condition, and any adjuvant therapy such as chemotherapy and/or radiotherapy, etc. The patients were followed until August 31, 2017 or until they died.

### Statistical analysis

Receiver operating characteristic (ROC) curve was performed to obtain the best cut-off value for monocyte count to stratify patients at a high risk of tumor recurrence, distant metastasis, or death. In the ROC curve, the point with the maximum sensitivity and specificity was selected as the best cut-off value. Disease-free survival (DFS) was calculated from the date of surgery to the date of recurrence/metastasis or death with any cause, and overall survival (OS) was presented from the date of surgery to the date of death with any cause. Fisher’s exact test or χ2-test for categorical variables and t-test for continuous variables were used to analyze the clinicopathological features for the two groups divided by the cut-off value of monocyte. A life table was made to calculate 1, 3 and 5-year survival rate. Survival curves were plotted using the Kaplan–Meier method and compared using the log-rank test. The prognostic factors of OS and DFS were analyzed by Cox proportional hazard model with univariate and multivariate analysis. Factors significant in the univariate analysis were included in the multivariate analysis. Subgroup analysis was used to further discriminate tumor prognosis between monocyte count and other prognostic factors: pathological stage, histological type, etc. All *P* values were two-tailed with less than 0.05 considered to be statistically significant. Statistical analysis was performed using SPSS (SPSS version 19.0, Chicago, IL, USA) and STATA 14.0 (STATA Corporation, College Station, TX, USA). Survival curves were drawn by GraphPad Prism 5.0 (GraphPad Software, San Diego, CA).

## Results

### Retrospective study selection process

**Fig. 1:**
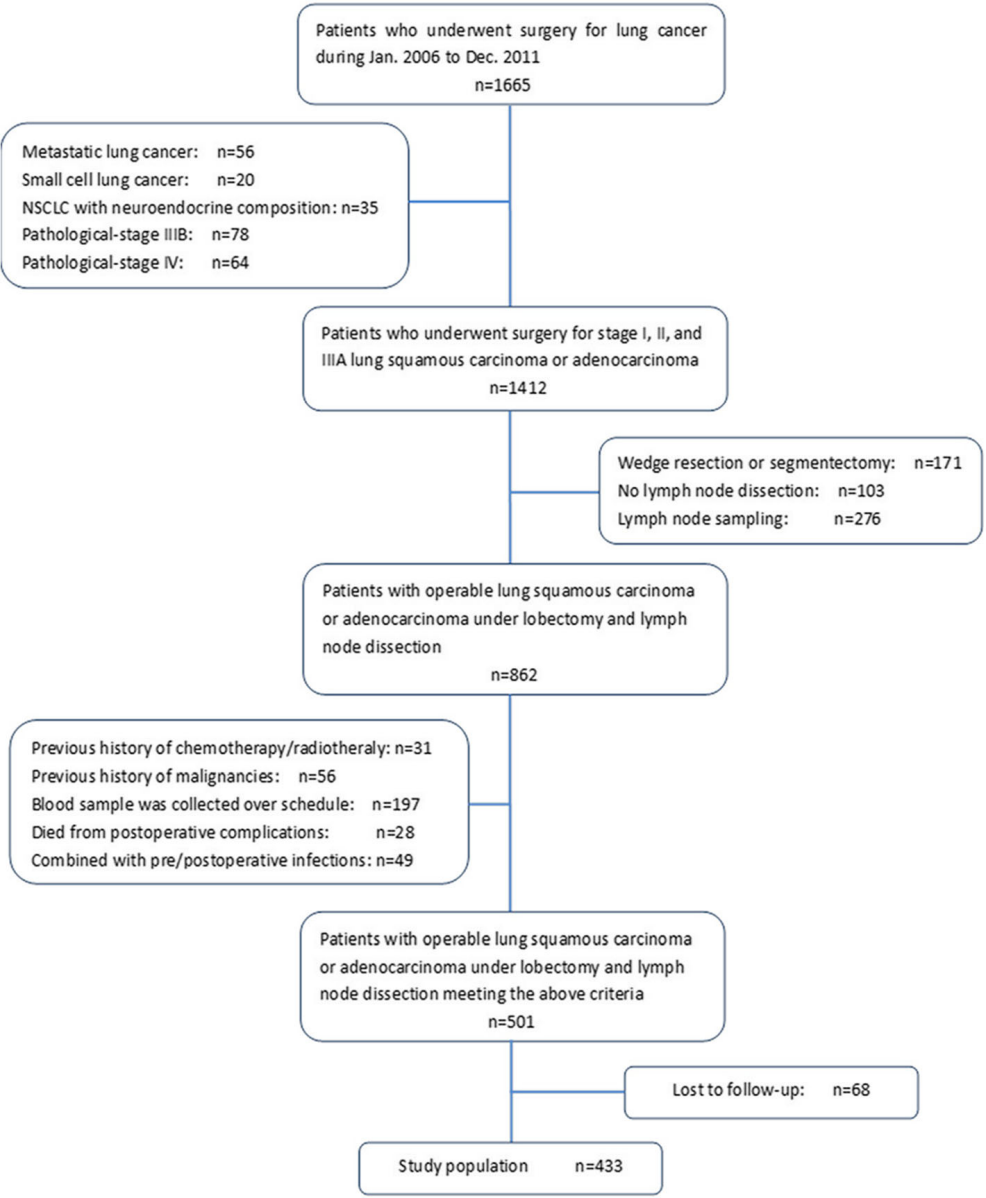
Flow chart of patient selection

The selection process of patients was shown in Fig. 1. There were 1665 patients identified in total. 1232 patients were excluded for cancer type, pathological stage, or surgical procedure mismatch, previous history of malignancies or chemotherapy/radiotherapy, and those co-morbid with infections. There were 433 patients enrolled in our analysis. The average age and standard deviation for the 433 patients were 60.6 and 10.0, respectively. Among them, there were 278(64.2%) males and 155(35.8%) females. Video-assisted thoracic surgery (VATS) was performed in 221(51.0%) patients compared with traditional thoracotomy surgery in 212(49.0%) patients. Specimens were histologically proven to be lung adenocarcinoma in 264(61.0%) patients and squamous cell carcinoma in 169(39.0%) patients. Details are presented in Table 1.

**Table 1:** Basic characteristics of clinicopathological features of patients with different monocyte counts

Category	N (%)	Preoperative monocyte (10^9^/L)		Postoperative monocyte (10^9^/L)	
		< 0.375	≥0.375	< 0.845	≥0.845
Gender					
Male	278(64.2%)	122	156	155	123
Female	155(35.8%)	128	27	116	39
Age	433(100%)	61.08 ± 9.74	59.9 ± 10.47	60.2 ± 10.05	61.21 ± 10.08
Smoking history					
Yes	258(59.7%)	115	143	148	110
No	174(40.3%)	135	39	123	51
Location					
Left superior	128(29.6%)	74	54	80	48
Left inferior	62(14.3)	39	23	40	22
Right superior	128(29.6%)	79	49	80	48
Right middle	44(10.2)	24	20	24	20
Right inferior	71(16.4%)	34	37	47	24
Procedure					
VATS	221(51%)	139	82	148	73
Thoracotomy	212(49%)	111	101	123	89
T stage					
T1	84(19.4%)	58	26	50	34
T2	279(64.4%)	161	118	178	101
T3	55(12.7%)	26	29	31	24
T4	15(3.5%)	5	10	12	3
N stage					
N0	268(62.0%)	158	110	184	84
N1	71(16.4%)	35	36	42	29
N2	93(21.5%)	56	37	45	48
Stage					
I	205(47.3%)	126	79	139	66
II	107(24.7%)	59	48	68	39
IIIA	121(27.9%)	65	56	64	57
Histological type					
Adenocarcinoma	259(59.8%)	181	78	168	91
Squamous carcinoma	174(40.2%)	69	105	103	71
Differentiation					
Well	11(2.6%)	8	3	9	2
Moderate	261(61.7%)	155	106	159	102
Poor	151(35.7%)	80	71	98	53

*T stage* tumour, *N stage* node, *VATS* Video-assistant thoracoscopic surgery, *N* cases sample size

^a^Significance of Fisher’s exact test, X^2^-test or t-test. *P* < 0.05 was considered statistically significant

### Cut-off selection for monocyte counts and survival rate calculation

**Fig. 2:**
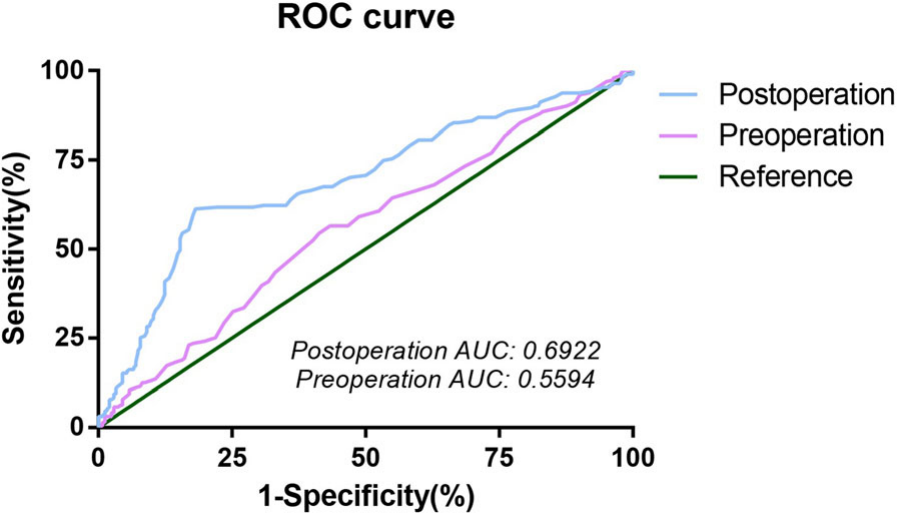
Receiver operating characteristic curve for determination of the cut-off value for monocyte. The cut-off value for preoperative monocyte count was 0.375*10^9^/L with area under the curve (AUC) of 0.566; while 0.845*10^9^/L with AUC of 0.692 for postoperative monocyte count

By analyzing the ROC curve, the cut-off value for preoperative monocyte count was 0.375*10^9^/L with the area under the curve (AUC) of 0.566; while for postoperative monocyte count, the cut-off value was 0.845*10^9^/L with AUC of 0.692 (Fig. 2). The baseline characteristics were stratified by high versus low preoperative monocyte and early postoperative monocyte count (Table 1).

We then performed the Kaplan-Meier method to identify difference of survival rates between two groups stratified by monocyte count (low vs. high) of both preoperative and early postoperative group.

### Preoperative group OS and DFS

The 1-year OS of low and high subgroups were 95% and 93% (*P* = 0.003), and the 1-year DFS of low and high subgroups were 82% and 85% (*P* = 0.106). The 3-year OS of low and high subgroups were 82% and 74% (*P* = 0.003), and the 3-year DFS of low and high subgroups were 67% and 60% (*P* = 0.106), respectively. Additionally, the 5-year OS of low and high subgroups were 72% and 58% (*P* = 0.003), and the 5-year DFS of low and high subgroups were 62% and 51% (*P* = 0.106). However, when stratified by gender, age, smoking history, stage, T stage, N stage and surgical procedure, no statistical significance were seen in subgroup study of the preoperative group.

### Early postoperative group OS and DFS

**Fig. 3:**
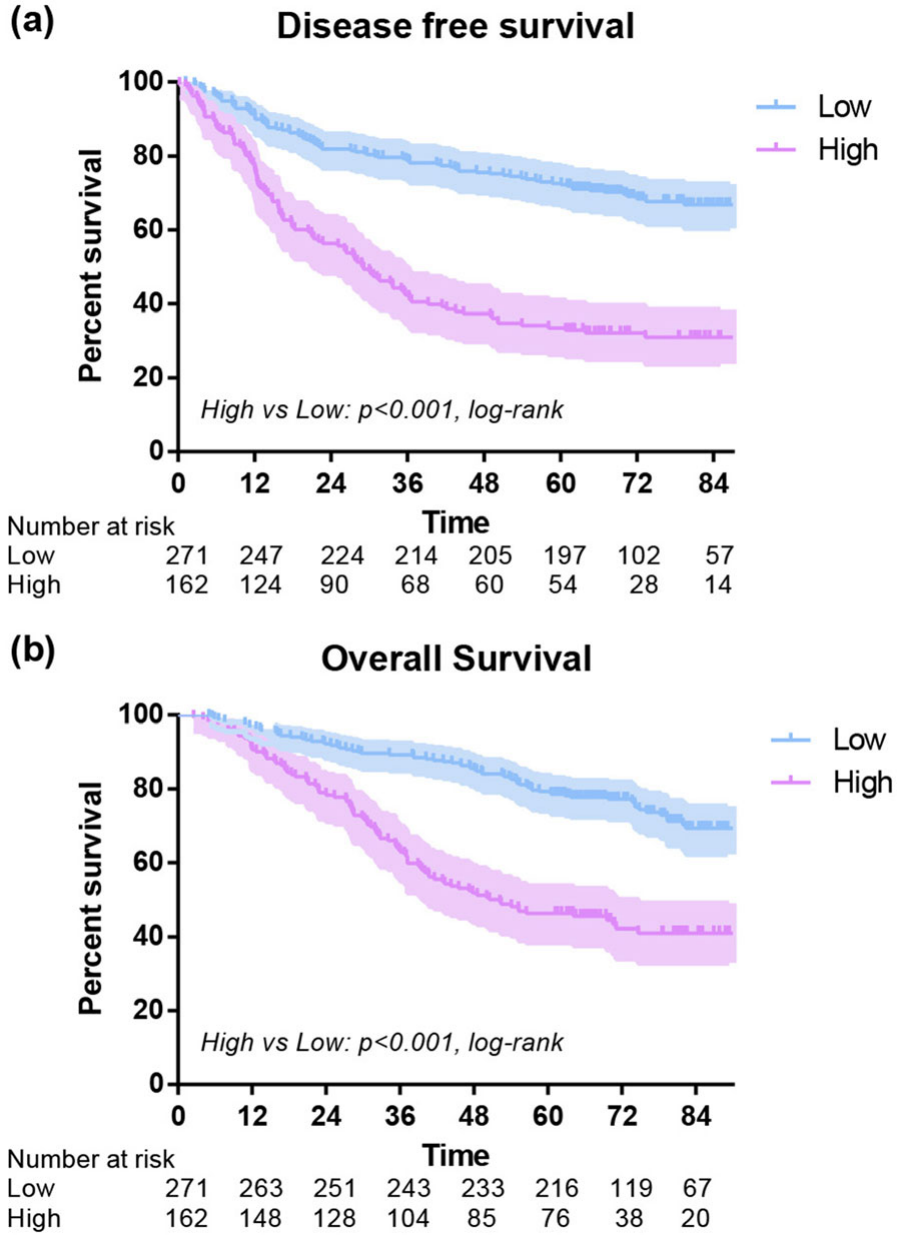
Disease-free survival (**a**) and overall survival (**b**) of high and low postoperative monocyte level

**Fig. 4:**
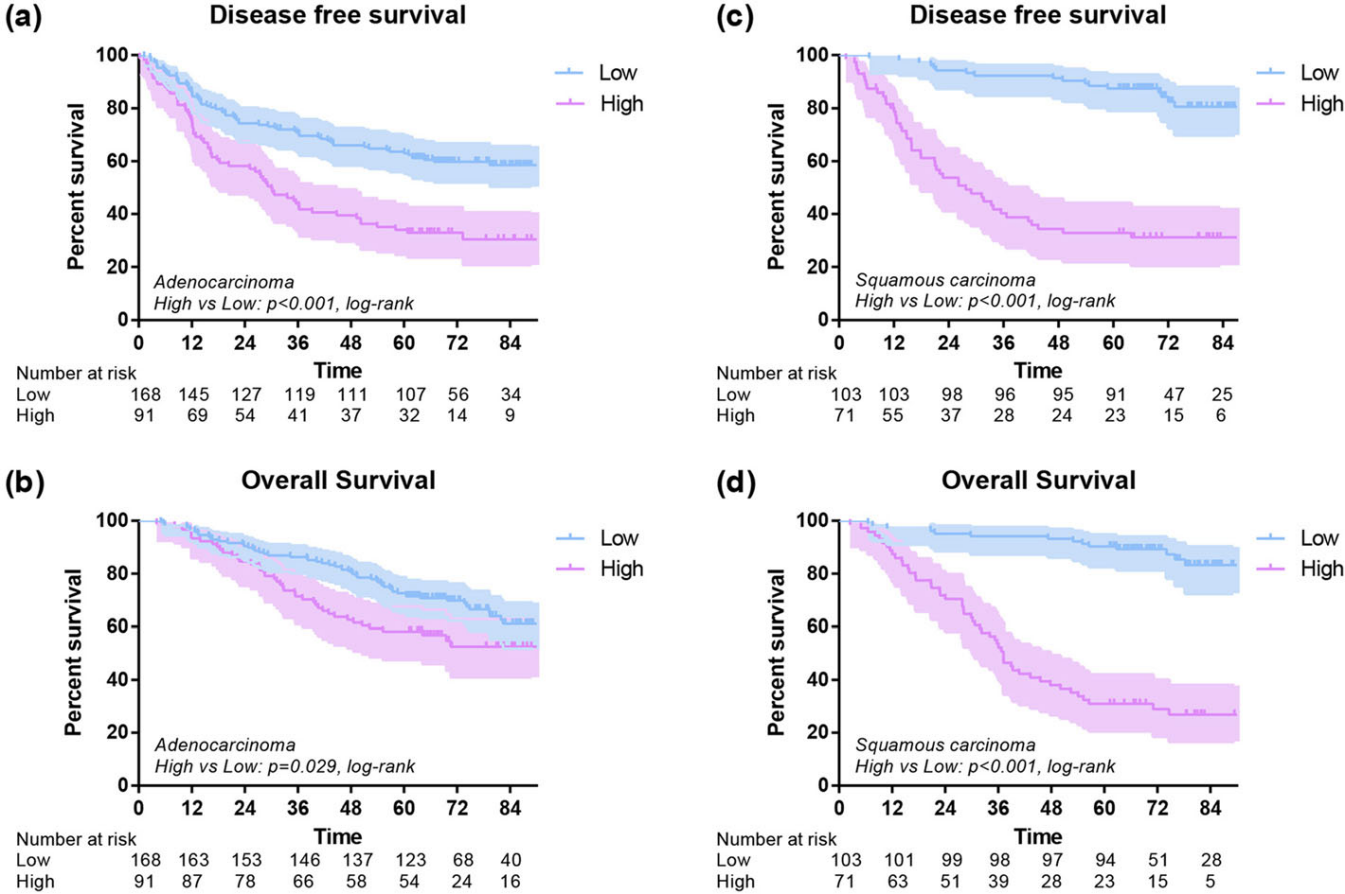
Disease-free survival and overall survival of 433 lung cancer patients with different monocyte stratified by histological type of adenocarcinoma (**a**, **b**) and squamous cell carcinoma (**c**, **d**)

**Fig. 5:**
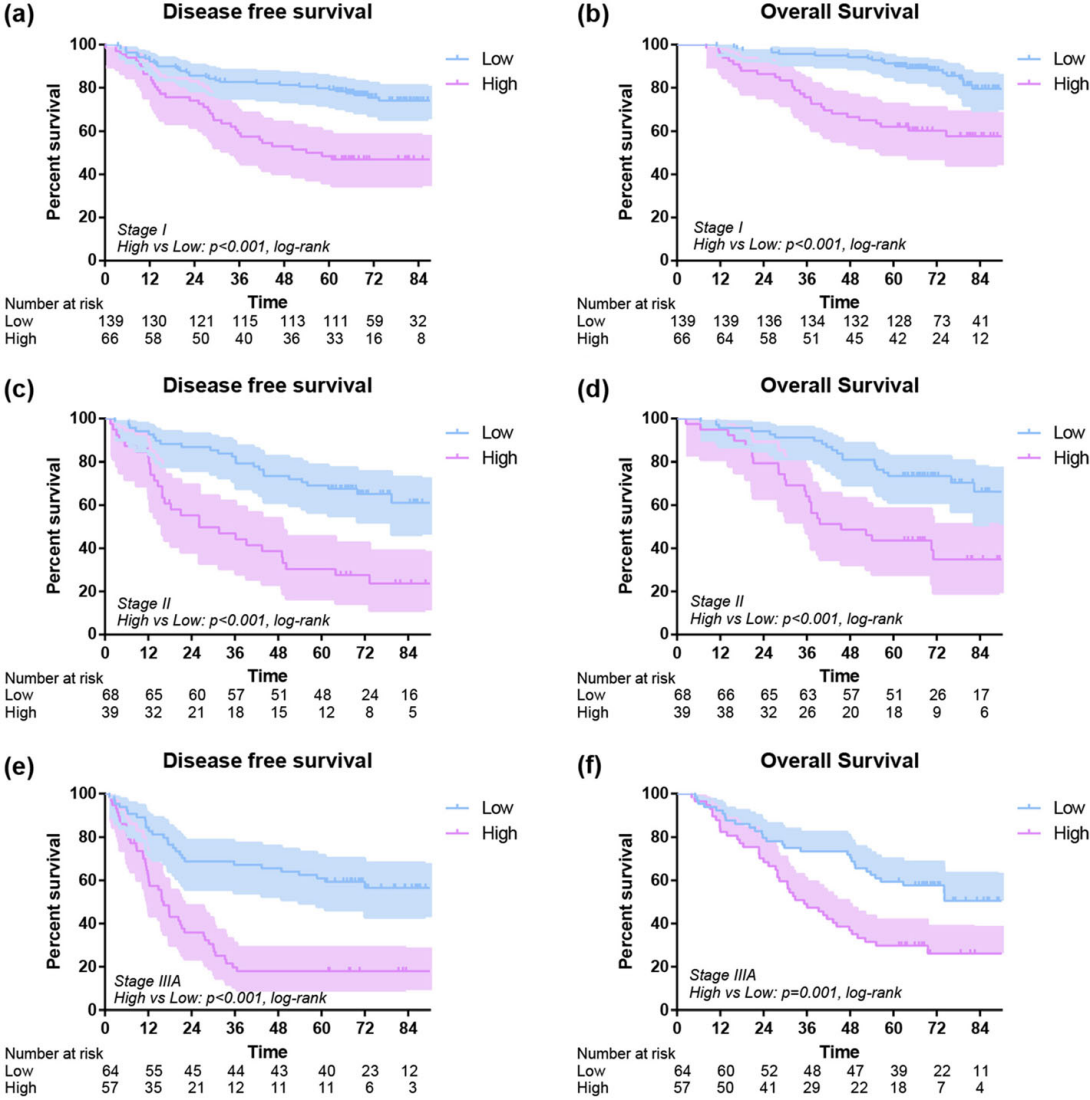
Disease-free survival and overall survival of 433 lung cancer patients with different monocyte stratified by tumor stage(stage I disease: **a**, **b**; stage II disease: **c**, **d**; stage IIIA disease: **e**, **f**)

**Fig. 6:**
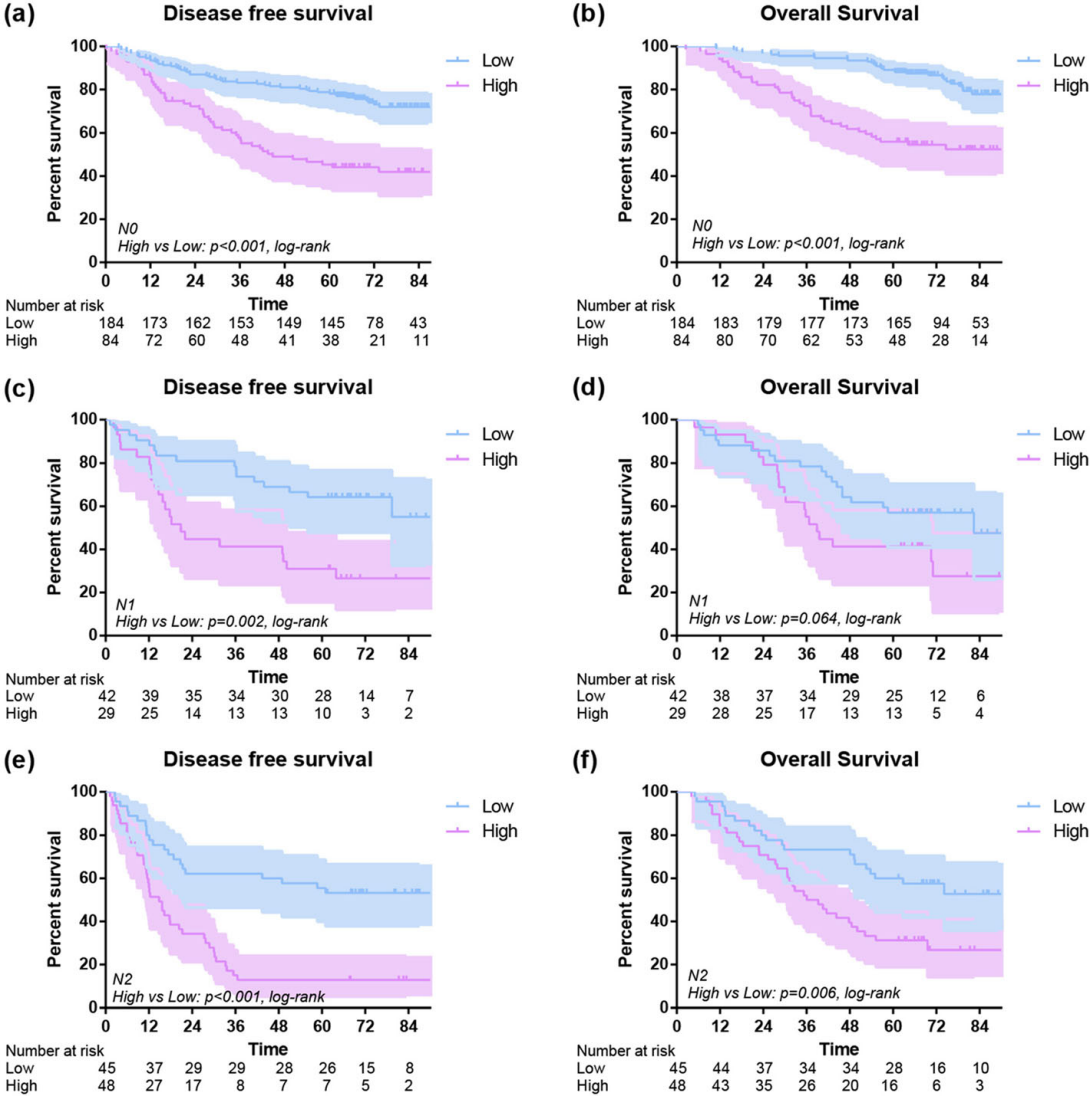
Disease-free survival and overall survival of 433 lung cancer patients with different monocyte stratified by N stage(N0 stage: **a**, **b**; N1 stage: **c**, **d**; N2 stage: **e**, **f**)

The 1-year OS of low and high subgroups were 96% and 90% (*P* = 0.004), and the 1-year DFS of low and high subgroups were 90% and 71% (*P* < 0.001). The 3-year OS of low and high subgroups were 89% and 62% (*P* < 0.001), and the 3-year DFS of low subgroup was 78% compared to 41% in high group (*P* < 0.001). In addition, the 5-year OS of low and high subgroups were 79% and 46% (*P* < 0.001), and the 5-year DFS of low and high subgroups were 72% and 33% (*P* < 0.001). Overall, an early postoperative elevated monocyte count was significantly associated with poor OS (*P* < 0.001) and DFS (*P* < 0.001) (Fig. 3). In further analysis, high level postoperative monocyte count was associated with poor OS and DFS in both adenocarcinoma and squamous carcinoma subgroups (Fig. 4). These differences were also significant in subgroup analysis when stratified by gender, age, smoking history, stage, T stage, N stage and surgical procedure in postoperative group (Figs. 5, 6).

### Univariate analysis

Univariate prognostic analysis was performed to detect the prognostic significance of clinicopathological factors, and preoperative and early postoperative blood monocyte count (Table 2). N stage (*P* < 0.001), tumor stage (*P* < 0.001), histological type (*P* = 0.014), preoperative (*P* = 0.023) and postoperative monocyte count level (*P* < 0.001) were significantly associated with DFS, while age (*P* = 0.017), surgery procedure (*P* = 0.002), T stage (*P* = 0.003), N stage (*P* < 0.001), tumor stage (*P* < 0.001), preoperative(*P* = 0.024) and postoperative monocyte count level (*P* < 0.001) showed significant relationships with OS.

**Table 2:** Univariate analysis of clinicopathological parameters and inflammatory biomarkers influencing prognosis

Category	Variables	Disease-free survival		Overall survival	
		HR (95% CI)	*P* ^a^	HR (95% CI)	*P* ^a^
Gender	Male	Reference		Reference	
	Female	0.963(0.716–1.295)	0.801	0.841(0.581–1.216)	0.358
Age	Per year	1.008(0.993–1.022)	0.311	1.023(1.004–1.042)	**0.017**
Smoking history	Yes	Reference		Reference	
	No	1.009(0.756–1.348)	0.950	0.838(0.585–1.202)	0.337
Location	Left superior	Reference		Reference	
	Left inferior	0.989(0.634–1.545)	0.962	1.492(0.870–2.560)	0.146
	Right superior	0.875(0.604–1.267)	0.479	1.205(0.758–1.916)	0.431
	Right middle	1.263(0.785–2.031)	0.335	1.486(0.817–2.700)	0.194
	Right inferior	0.655(0.410–1.047)	0.077	0.899(0.506–1.596)	0.715
Procedure	VATS	Reference		Reference	
	Thoracotomy	1.455(1.093–1.937)	**0.010**	1.760(1.236–2.505)	**0.002**
T stage	T1	Reference		Reference	
	T2	1.219(0.833–1.783)	0.309	1.268(0.775–2.072)	0.344
	T3	1.559(0.945–2.573)	0.082	2.453(1.361–4.421)	**0.003**
	T4	1.376(0.609–3.111)	0.443	1.602(0.601–4.271)	0.346
N stage	N0	Reference		Reference	
	N1	2.467(1.695–3.592)	**< 0.001**	2.869(1.833–4.491)	**< 0.001**
	N2	3.349(2.414–4.647)	**< 0.001**	3.442(2.306–5.139)	**< 0.001**
Stage	I	Reference		Reference	
	II	1.847(1.273–2.679)	**0.001**	2.342(1.460–3.757)	**< 0.001**
	IIIA	3.163(2.260–4.428)	**< 0.001**	4.078(2.658–6.258)	**< 0.001**
Histological type	Squamous carcinoma	Reference		Reference	
	Adenocarcinoma	2.994(1.418–6.113)	**0.014**	1.465(0.712–3.018)	0.300
Differentiation	Well	Reference		Reference	
	Moderate	6.446(0.899–46.191)	0.064	3.869(0.538–27.831)	0.179
	Poor	7.172(0.996–51.643)	0.050	4.340(0.599–31.455)	0.146
Preoperative monocyte	< 0.375	Reference		Reference	
	≥0.375	1.391(1.047–1.849)	**0.023**	1.490(1.053–2.109)	**0.024**
Postoperative monocyte	<0.845	Reference		Reference	
	≥0.845	3.974(2.956–5.342)	**< 0.001**	3.826(2.662–5.499)	**< 0.001**

*T stage* tumor, *N stage* node, *VATS* Video-assistant thoracoscopic surgery, *HR* hazard ratio, *CI* confidence interval

^a^*P* value of univariate analysis. *P* < 0.05 was considered statistically significant

The bold data means the data has statistically significance

### Multivariate analysis

The clinicopathological factors proved to be prognostic predictors in univariate analysis were included as covariates in further multivariate analysis (Table 3). Finally, thoracotomy (HR = 1.520, 95%CI: 1.117–2.069, *P* = 0.008), positive N status (HR = 2.506, 95%CI: 1.625–3.864, P < 0.001), Adenocarcinoma (HR = 2.273, 95%CI: 1.181–4.374, *P* = 0.014) and high postoperative monocyte count (HR = 2.991, 95%CI: 2.243–3.988, *P* < 0.001) were risk factors with statistical significance for DFS. Correspondingly, age (HR = 1.022, 95%CI: 1.005–1.038, *P* = 0.009), thoracotomy (HR = 1.700, 95%CI: 1.163–2.486, *P* = 0.006), advanced tumor stage (HR = 2.253, 95%CI: 1.178–4.309, *P* < 0.001), and high postoperative monocyte count (HR = 2.705, 95%CI: 1.977–3.700, *P* < 0.001) were observed as risk factors for OS. However, preoperative monocyte was no more associated with either DFS or OS within multivariate analysis.

**Table 3:** Multivariate analysis of clinicopathological parameters and inflammatory biomarkers influencing prognosis

Category	Variables	HR (95% CI)	*P* ^a^
Disease-free survival			
Procedure	VATS	Reference	
	Thoracotomy	1.520(1.117–2.069)	**0.008**
N stage	N0	Reference	
	N1	2.319(1.584–3.396)	**< 0.001**
	N2	2.584(1.845–3.620)	**< 0.001**
Histological type	Squamous carcinoma	Reference	
	Adenocarcinoma	2.273(1.181–4.374)	**0.014**
Postoperative monocyte	<0.845	Reference	
	≥0.845	3.684(2.729–4.975)	**< 0.001**
Overall survival			
Age	Per year	1.034(1.014–1.054)	**0.001**
Procedure	VATS	Reference	
	Thoracotomy	1.700(1.163–2.486)	**0.006**
Stage	I	Reference	
	II	2.228(1.383–3.592)	**0.001**
	IIIA	3.592(2.327–5.546)	**< 0.001**
Postoperative monocyte	<0.845	Reference	
	≥0.845	3.403(2.362–4.902)	**< 0.001**

*N stage* node, *VATS* Video-assistant thoracoscopic surgery, *HR* hazard ratio, *CI* confidence interval

^a^*P* value of multivariate analysis. *P* < 0.05 was considered statistically significant

The bold data means the data has statistically significance

## Discussion

Immune response is an essential component of tumor progression. Studying the immune factors expressed in the tumor microenvironment can further stratify the prognosis of cancer [10]. Back in 1997, Negus et al. had demonstrated that a number of cells expressing chemokine receptors could infiltrate to tumor areas [11]. Since then, various experimental studies have elucidated identities of these chemokines. Inflammatory markers such as C-reactive protein in esophageal squamous cancer [12], Colony-stimulating factor-1 in mammary tumor [13], and inhibitors of metalloproteinases in NSCLC [14], all have been suggested as alternative markers for tumor progression [8]. Meanwhile, the peripheral blood cell counts, an easily assessible biomarker, has also been suggested as predictors of tumor prognosis. An elevated neutrophil, monocyte and leukocyte counts were proven to be associated with poor survival in patients with metastatic melanoma [15].

### High pre-operative monocyte count associated with poor survival following surgical procedures

Monocytes belonged to circulating peripheral blood cells that played the crucial role in immune response with the capability of differentiating into macrophages and antigen-presenting cells (APCs). Thus, they formed the first line of innate immune defense [16]. In addition, monocytes could activate T and B lymphocytes and further produce cytokines such as IL-12 and TNF-α to stimulate the immune response [17]. However, either overstimulation or immunosuppression of monocytes caused by surgical procedure and/or other external factors could disrupt the immune system [18, 19]. Previous studies have shown an elevated preoperative monocyte count to be a poor prognostic factor in esophageal squamous cell carcinoma, mantle cell lymphoma, follicular lymphoma, and classical Hodgkin lymphoma, respectively [6, 7, 20, 21]. During the early postoperative period, absolute monocyte count is increased but monocyte function is impaired, as demonstrated by a decreased ability to synthesize IL-12 and TNF-α, to express HLA-DR, and to act as the APC [22]. To the best of our knowledge, this is the first study suggesting the prognostic significance of the early postoperative monocyte count in patients with lung adenocarcinoma or squamous cell carcinoma after lobectomy.

### Postulated pathophysiologic mechanisms of monocyte proliferation in tumors

The mechanism of elevated monocyte count and poor prognosis of several kinds of tumors is still unclear, despite several proposed postulations. First, it was hypothesized that monocytes are attracted by several cytokines or chemokines to the tumor site and then differentiated into tumor-associated macrophages (TAMs), which further promoted those invading leukocytes to bring out potentials of angiogenesis, motility, and invasion [23, 24]. Angiogenic signals from surrounding cells resulted in vasodilatation and increased vascular permeability [25, 26], forming a vicious cycle for tumor progression. Second, human monocyte subsets were differentiated according to their surface CD14/CD16 expression as “majority/classical” (CD14++CD16-), “minority/non-classical” (CD14 + CD16+) and the subset with pro-angiogenic feature (CD14++CD16 + CCR2+) [27, 28]. Among all subsets of the monocytes, the “majority/classical” accounts for approximately 90% of monocytes in healthy people [29]. However, Schauer et al. found that along with the increased number of monocytes, the major monocyte population shifted from CD16- to CD16+ after liver resection, showing a stronger potential of angiogenesis [30].

### Macrophages types M1 and M2 and monocytes in post-operative patient

It is known that classical monocytes are recruited to tumor sites, contributing to tumor macrophage content and promoting tumor growth and metastasis [31]. Meanwhile, Hanna et al. also found out the potential protective role of nonclassical “patrolling” monocytes on tumor growth and metastasis [32]. This was also our next step to find out whether it will happen after lobectomy of lung cancer and what the exact type was and the alternatives of the function in immunity. Third, peripheral monocytes grow into TAMs when entering tumor areas. TAMs are classified into two phenotypes: M1 and M2. Activated M1 macrophages have the anti-tumor response, while M2 macrophages, activated by tumor-derived cytokines, were suitable for tumor development [33, 34]. A previous study has reported that circulating macrophages predict tumor recurrence after surgery in patients with NSCLC [35], while in this study, an elevated early postoperative peripheral monocyte count was significantly associated with a poor prognosis in patients with lung adenocarcinoma or squamous cell carcinoma after lobectomy. These results suggest a complex association between peripheral TAMs (M2 type) and monocytes, though further studies are needed to verify this. Moreover, the reason why we focused on the early postoperative period - the most turbulent stage of host immune system caused by surgical trauma and/or tumor removal effect - was that we hypothesized that immune environment changes in the early post-op period might relate with tumor progression more closely than that of the preoperative period.

### Present study associates elevated early postoperative monocyte counts with poor survival

In the present study, an elevated early postoperative monocyte count was shown to predict a poor DFS and OS both in the univariate and multivariate analysis. In addition, as shown in the subgroup analysis, the monocyte count was found to be significantly associated with poor prognosis when stratified by gender, age, smoking history, TNM stage, surgical procedure and histological type. All of the above indicated that an elevated early postoperative blood monocyte count to be a very strong prognostic factor in tumor progression.

### Limitations of current study

Limitations of the current study are inherent to its design, including the retrospective data collection and several confounding factors when comparing postoperatively. For example, a t-test performed from Table 1 shows an association of male gender with a high post-operative monocyte count. Similarly, smoking is associated with a high post-operative monocyte count. However, since male smokers outnumber female smokers in China [36], and most of the patients in our lung cancer clinic are male smokers, there is no way to untangle these two factors in a retrospective study. Or perhaps, an unidentified factor may influence both male gender and smoking independently to cause monocyte count increase. A prospective study may be able to control for these confounders. Moreover, the small number of patients, especially the cases with endpoints, also limited the conclusion of the current study.

### Cautious and prudent monitoring

Although there is no evidence currently to change the management of patients following a lobectomy, we are reporting some prognostic significance of the postoperative monocyte count on survival. The increased mortality for patients with elevated early postoperative monocyte, particularly in patients with squamous cell carcinoma, warrants a cautious approach to monitor disease progression.

## Conclusion

The present study supported the prognostic significance of early postoperative peripheral blood monocyte count in patients with operable lung adenocarcinoma or squamous cell carcinoma after lobectomy in both OS and DFS. This easily measured blood parameter may provide useful information for the clinicians to stratify patients. Further investigations will be needed to figure out the oncological significance of monocyte and its subsets, and the association with the host inflammatory microenvironment.

## Abbreviations

APCs: Antigen-presenting cells
AUC: Area under the curve
DFS: Disease-free survival
IL: Interleukin
MRI: Magnetic resonance imaging
NSCLC: Non-small cell lung cancer
OS: Overall survival
PET-CT: Positron emission tomography scan and CT
ROC: Receiver operating characteristic
TAMs: Tumor-associated macrophages
TNF: Tissue necrosis factor
VATS: Video-assisted thoracic surgery
